# Supplementary material for: Tunable and parabolic piezoelectricity in hafnia under epitaxial strain
Source: Nat Commun. 2024 Jan 9;15:394. doi: 10.1038/s41467-023-44207-w (PMC10776838; doi:10.1038/s41467-023-44207-w)
Supplement: Supplementary file 1 — Supplementary Information [file 41467_2023_44207_MOESM1_ESM.pdf]

Supplementary Information

Tunable and parabolic piezoelectricity in hafnia under epitaxial strain

Hao Cheng[1,2†], Peijie Jiao[1,2†], Jian Wang[1,2], Mingkai Qing[1,2], Yu Deng[1,2], Jun-Ming Liu[1], Laurent Bellaiche[3*], Di Wu[1,2*], Yurong Yang[1,2*]

[1]*Laboratory of Solid State Microstructures, Nanjing University, Nanjing 210093, China*

[2]*Jiangsu Key Laboratory of Artificial Functional Materials, Department of Materials Science and Engineering, Nanjing University, Nanjing 210093, China*

[3]*Physics Department, Institute for Nanoscience and Engineering, University of Arkansas, Fayetteville, Arkansas 72701, USA*

Contents:

I. The methods of applying external electric field

II. Piezoelectric response for (111)-oriented hafnia on (110)-oriented substrates

III. Phase stability and piezoelectric properties for (111)-oriented hafnia on (001)-oriented substrates

IV. Piezoelectric response of $Hf_{0.5}Zr_{0.5}O_2$ for (111)-oriented hafnia on (110)-oriented substrates

Reference

[†] These authors contributed equally to this work.

* Corresponding author. Email: laurent@uark.edu, diwu@nju.edu.cn, yangyr@nju.edu.cn

I. The methods of applying external electric field

To determine the response of structure and properties to finite external electric field, we used the scheme of electric enthalpy functional

$$F(\mathbf{R},\mathcal{E}) = E^0_{KS}(\mathbf{R}) - \mathbf{P}(\mathbf{R})\cdot\mathcal{E}, \qquad \text{(S1)}$$

where $E^0_{KS}(\mathbf{R})$ is the zero-field ground-state Kohn-sham energy at the coordinates **R**, and **P** is the polarization. $\mathcal{E}$ is the electric field which can be DC field, and also can be an AC sinusoidal electric field $\mathcal{E}(t) = \mathcal{E}_{max}\sin(2\pi\omega t + \pi)$ with a frequency ω of 10 GHz and a magnitude electric field $\mathcal{E}_{max}$ of 4 MV/cm or 8 MV/cm. In the presence of an applied electric field, the equilibrium coordinates that minimize the electric enthalpy function should satisfy the force-balance equation. $-\frac{dE^0_{KS}}{dR} + Z^0\cdot\mathcal{E} = 0$, where Z^0 is the zero-field Born effective change tensor. Such scheme had been shown to provide good accuracy for electric-field-related physical responses in ferroelectric and multiferroic compounds[1,2]. Note, however, that theoretical ab-initio fields can typically be considered to be larger than experimental ones by one or two orders of magnitudes[3,4], likely because of the fact that structural defects are typically omitted in the calculations. It is also important to realize that electric fields as high as $\simeq$ 5 MV/cm have been recently experimentally achieved in some multiferroic/ferroelectric films[5], which is of the same order than the largest fields applied in the present work.

II. Piezoelectric response for (111)-oriented hafnia on (110)-oriented substrates

Figure S1 shows energies of different phases with epitaxial strain of (111)-oriented hafnia. For tensile strains larger than 1.5%, the energy of the $P2_1/c$-like phase is the lowest, meanwhile, the energy difference between the $P2_1/c$-like phase and the $Pca2_1$-like phase in thin films is comparable to that of the bulk, which is similar to the recent calculations of Ref. [6].

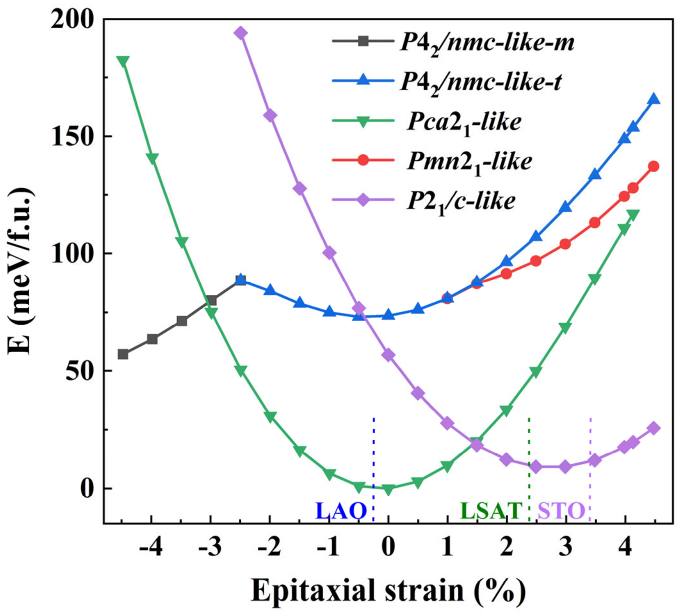

Figure S1. Energies of different phases (including monoclinic phase $P2_1/c$-like) as a function of the epitaxial strain.

Figure S2 shows the piezoelectric response of (111)-oriented hafnia under electric field for an epitaxial strain of 0.9%. It is found that the out-of-plane strain increases when the electric field increases or decreases from -0.03 eV/ Å. Under electric fields smaller than -0.03 eV/ Å (antiparallel to the polarization), the lattice constant expands, implying a negative piezoelectric effect. Under electric fields larger than -0.03 eV/ Å, the lattice constant expands, implying positive piezoelectric effect. This converse

piezoelectric effect can be described by

$$\varepsilon_3 = e_{33}^{*} E_3 + B_{333}^{*} E_3^2 \qquad \text{(S2)}$$

where ε is the strain, number 3 indicates the out-of-plane direction, E is the electric field, ε is the strain, e^{*} is the linear converse piezoelectric coefficient, and B^{*} is the quadratic converse piezoelectric coefficient. As shown in Fig. S1, the equation above fits very well the strain-versus-electric field curve, with e_{33}^{*} = 0.006 Å/V and B_{333}^{*}=0.106 Å^2/V^2.

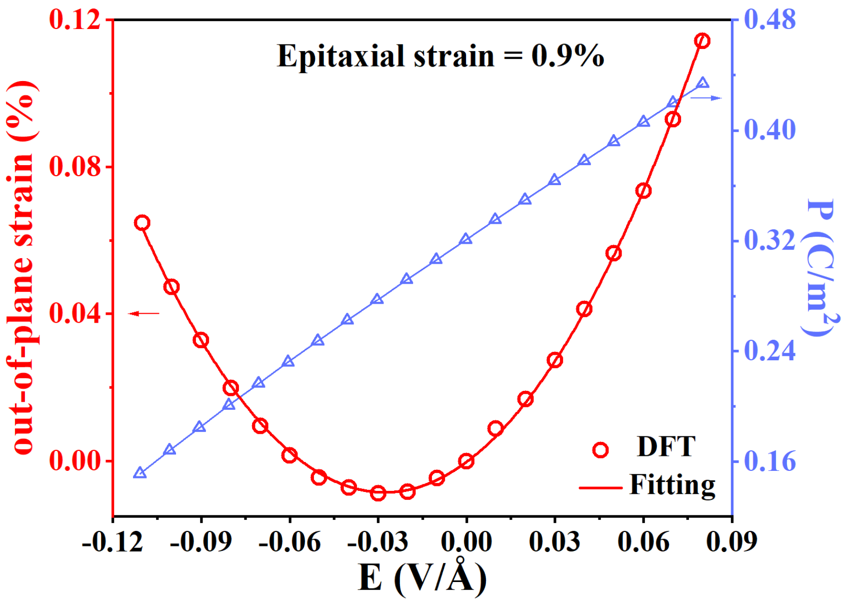

Figure S2. The out-of-plane strain and polarization (along the [111] direction) as a function of the electric field for an epitaxial strain of 0.9%. The polarization linearly increases with the increase of electric field. The out-of-plane strain rather shows a parabolic behavior with respect to electric field. The lattice constant is the smallest at the electric field of about -0.03 V/Å. At electric fields larger than -0.03 V/Å, the positive piezoelectricity induces an increase of the out-of-plane strain when increasing the electric field parallel to polarization. At electric fields smaller than -0.03 V/Å, the negative piezoelectricity leads to the increase of the out-of-plane strain when increasing the negative electric field antiparallel to the polarization.

III. Phase stability and piezoelectric response for (111)-oriented hafnia on (001)-oriented substrates

Figure S3 shows the possible lattices matchings between (111)-oriented hafnia and (001)-oriented substrates. The strains of three possible matchings on substrates (001)-oriented STO, LSAT and LAO are summarized in Table S1. As the smaller lattice vectors of (111) hafnia films matching the substrate induce smaller energy between the interface, the vector of $a \approx 3a_0$ with in-plane lattice angle γ=120.5° form the most possible matching patten with substrates. We use these lattice vectors with γ=120.5° to investigate the piezoelectric response.

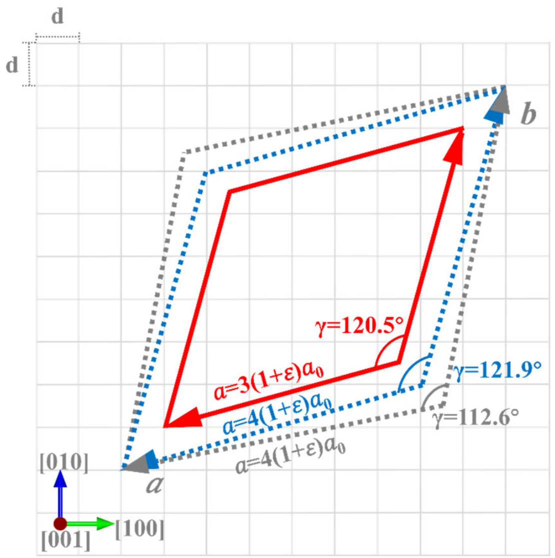

Figure S3. Sketch of lattices matching between (111)-oriented hafnia and (001)-oriented substrates. The gray grids give the substrate lattices. Red solid, blue dashed, and gray dashed vectors represent the possible lattice vectors of (111)-oriented hafnia. The in-plane lattice angle γ and lattice amplitude *a* are given.

Figure S4 shows the energies of different phases in (111)-oriented hafnia as a function of the epitaxial strain. One can confirm that the $Pca2_1$-like phase is the ground phase for epitaxial strains ranging from -3% to 4%, very similar to that (111)-oriented

hafnia on (110) substrates shown in Fig. 3a in the main text. Note that the (110) and (001) substrates give very different strains.

Table S1. Epitaxial strains of (111)-oriented HfO_2 on (001)-oriented substrates of $LaAlO_3$ (LAO), $(LaAlO_3)_{0.33}$-$(Sr_2AlTaO_6)_{0.67}$ (LSAT), and $SrTiO_3$ (STO). In the first column, a_0 represents the lattice constant (7.236 Å) of hafnia film at zero strain and the angle in parentheses represents the lattice in-plane angle γ. Comparing the strains, the vectors shown in red lines with $a \approx 3a_0$, γ=120.5° is the smallest lattice vector and have the small strain, which are the most possible experimental lattice vectors of (111)-oriented HfO_2 matching the (001) substrates.

	LAO	LSAT	STO
$3a_0$ (120.5°)	-1.0%	1.6%	2.6%
$4a_0$ (121.9°)	-5.2%	-2.7%	-1.7%
$4a_0$ (112.6°)	-0.4%	2.2%	3.3%

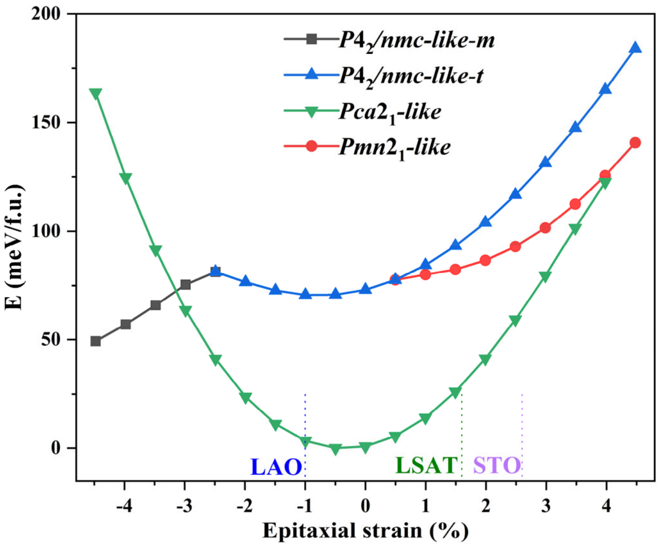

Figure S4. Energies of different phases in (111)-oriented hafnia as a function of the epitaxial strain. The blue, green, and purple dashed vertical lines represent the strains corresponding to (001)-oriented LAO, LSAT, and STO substrates, respectively. The strains labelled by dashed lines are computed using the red lattice vectors shown in Fig. S2.

Figure S5 shows the longitudinal linear piezoelectric coefficient e_{33} and quadratic coefficient B_{333} as a function of epitaxial strain. Similar to the (110) substrates described in the main text, e_{33} can be negative and positive depending on the amount of epitaxial strain. B_{333} is always negative and becomes large at large epitaxial strains. These linear and quadratic piezoelectric coefficients lead to linear, parabolic, and nonlinear piezoelectric behavior as discussed in the main text.

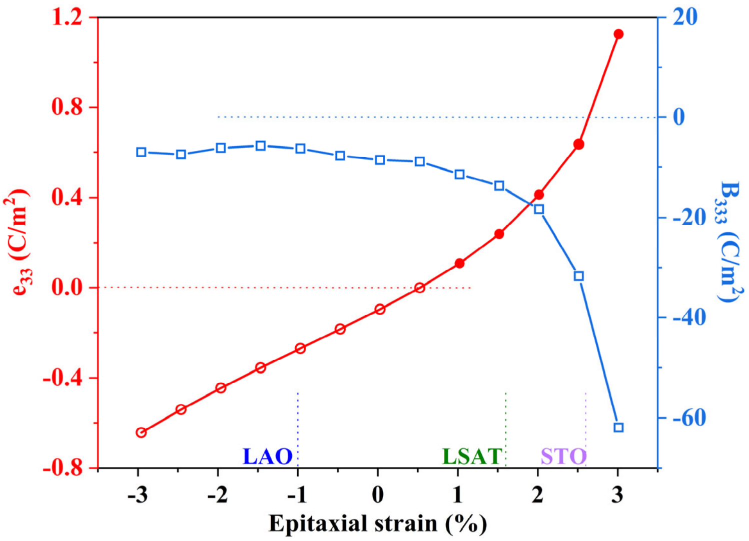

Figure S5. The linear coefficients e_{33} and quadratic coefficient B_{333} for (111)-oriented hafnia on (001)-oriented substrates as a function of epitaxial strain. The blue, green, and purple vertical dashed lines represent the strains corresponding to LAO, LSAT, and STO substrates, respectively.

IV. Piezoelectric response of $Hf_{0.5}Zr_{0.5}O_2$ for (111)-oriented hafnia on (110)-oriented substrates

Hf and Zr belong to the same column of the Periodic Table and thus have very similar chemical properties. In addition, due to the lanthanide contraction, the ion radii of Hf and Zr are very close to each other. Fig. S6 shows the piezoelectric behavior of $Hf_{0.5}Zr_{0.5}O_2$ which is similar to that of HfO_2 (see Fig. 2c in main text). The negative first-order piezoelectric coefficient at compressive strains and small tensile strains transforms into a positive coefficient at tensile strains larger than 1%. The second-order coefficient is always negative and becomes very large at tensile strains larger than 3%.

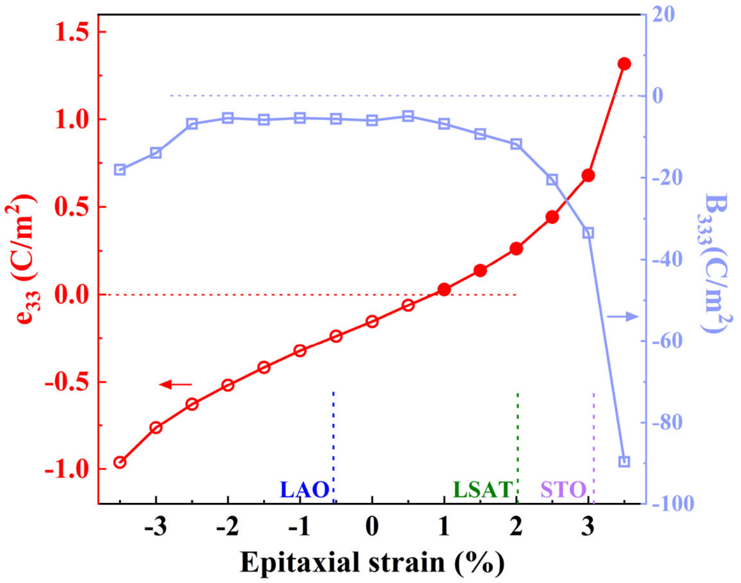

Fig. S6. The linear piezoelectric coefficients e_{33} and quadratic coefficient B_{333} of $Hf_{0.5}Zr_{0.5}O_2$ calculated by first-principles.

Reference

1 Fu, H. & Bellaiche, L. First-principles determination of electromechanical responses of solids under finite electric fields. *Phys. Rev. Lett.* **91**, 057601 (2003).

2 Chen, L., Yang, Y. & Meng, X. K. Giant electric-field-induced strain in lead-free piezoelectric materials. *Sci. Rep.* **6**, 25346 (2016).

3 Xu, B., Íñiguez, J. & Bellaiche, L. Designing lead-free antiferroelectrics for energy storage. *Nat. Commun.* **8**, 15682 (2017).

4 Jiang, Z. *et al.* Giant electrocaloric response in the prototypical Pb(Mg,Nb)O_3 relaxor ferroelectric from atomistic simulations. *Phys. Rev. B* **97**, 104110 (2018).

5 Chen, P. *et al.* Nonlinearity in the high-electric-field piezoelectricity of epitaxial $BiFeO_3$ on $SrTiO_3$. *Appl. Phys. Lett.* **100**, 062906 (2012).

6 Zhu, T., Deng, S. & Liu, S. Epitaxial ferroelectric hafnia stabilized by symmetry constraints. *Phys. Rev. B* **108**, L060102 (2023).
