# Supplementary material for: Tunable and parabolic piezoelectricity in hafnia under epitaxial strain
Source: Nat Commun. 2024 Jan 9;15:394. doi: 10.1038/s41467-023-44207-w (PMC10776838; doi:10.1038/s41467-023-44207-w)
Supplement: Supplementary file 3 — Reporting Summary [file 41467_2023_44207_MOESM3_ESM.pdf]

nature research

Corresponding author(s): Di Wu

Lasing Reporting Summary

Nature Research wishes to improve the reproducibility of the work that we publish. This form is intended for publication with all accepted papers reporting claims of lasing and provides structure for consistency and transparency in reporting. Some list items might not apply to an individual manuscript, but all fields must be completed for clarity.

For further information on Nature Research policies, including our data availability policy, see Authors & Referees.

• Experimental design

Please check: are the following details reported in the manuscript?

1. Threshold

Plots of device output power versus pump power over a wide range of values indicating a clear threshold

☐ Yes
☒ No

Our experiment focuses on the structures and piezoelectric properties of the prepared thin films, rather than using lasers to test the devices

2. Linewidth narrowing

Plots of spectral power density for the emission at pump powers below, around, and above the lasing threshold, indicating a clear linewidth narrowing at threshold

☐ Yes
☒ No

Our experiment focuses on the structures and piezoelectric properties of the prepared thin films, rather than using lasers to test the devices

Resolution of the spectrometer used to make spectral measurements

☐ Yes
☒ No

Our experiment focuses on the structures and piezoelectric properties of the prepared thin films, rather than using lasers to test the devices

3. Coherent emission

Measurements of the coherence and/or polarization of the emission

☐ Yes
☒ No

Our experiment focuses on the structures and piezoelectric properties of the prepared thin films, rather than using lasers to test the devices

4. Beam spatial profile

Image and/or measurement of the spatial shape and profile of the emission, showing a well-defined beam above threshold

☐ Yes
☒ No

Our experiment focuses on the structures and piezoelectric properties of the prepared thin films, rather than using lasers to test the devices

5. Operating conditions

Description of the laser and pumping conditions
Continuous-wave, pulsed, temperature of operation

☐ Yes
☒ No

Our experiment focuses on the structures and piezoelectric properties of the prepared thin films, rather than using lasers to test the devices

Threshold values provided as density values (e.g. W cm^{-2} or J cm^{-2}) taking into account the area of the device

☐ Yes
☒ No

Our experiment focuses on the structures and piezoelectric properties of the prepared thin films, rather than using lasers to test the devices

6. Alternative explanations

Reasoning as to why alternative explanations have been ruled out as responsible for the emission characteristics
e.g. amplified spontaneous, directional scattering; modification of fluorescence spectrum by the cavity

☐ Yes
☒ No

Our experiment focuses on the structures and piezoelectric properties of the prepared thin films, rather than using lasers to test the devices

7. Theoretical analysis

Theoretical analysis that ensures that the experimental values measured are realistic and reasonable
e.g. laser threshold, linewidth, cavity gain-loss, efficiency

☐ Yes
☒ No

Our experiment focuses on the structures and piezoelectric properties of the prepared thin films, rather than using lasers to test the devices

8. Statistics

Number of devices fabricated and tested

☐ Yes
☒ No

Our experiment focuses on the structures and piezoelectric properties of the prepared thin films, rather than using lasers to test the devices

Statistical analysis of the device performance and lifetime (time to failure)

☐ Yes
☒ No

Our experiment focuses on the structures and piezoelectric properties of the prepared thin films, rather than using lasers to test the devices
